# Virtual Disaster Simulation: Lesson Learned from an International Collaboration That Can Be Leveraged for Disaster Education in Iran

**DOI:** 10.1371/currents.dis.7007c0a03c4660f258994d3e150a033c

**Published:** 2015-07-13

**Authors:** Ali Ardalan, Joseph Kimuli Balikuddembe, Pier Luigi Ingrassia, Luca Carenzo, Francesco Della Corte, Ali Akbarisari, Ahmadreza Djalali

**Affiliations:** Disaster and Emergency Health Academy, National Institute of Health Research, School of Public Health, Tehran University of Medical Sciences, Tehran, Iran; Harvard Humanitarian Initiative, Harvard University, Cambridge, USA; Disaster and Emergency Health Academy, National Institute of Health Research, School of Public Health, Tehran University of Medical Sciences, Tehran, Iran; Research Center in Emergency and Disaster Medicine and Computer Science applied to Medicine(CRIMEDIM); Università del Piemonte Orientale, Novara, Italy; Research Center in Emergency and Disaster Medicine and Computer Science applied to Medicine (CRIMEDIM), Università del Piemonte Orientale, Novara, Italy; Research Center in Emergency and Disaster Medicine and Computer Science applied to Medicine (CRIMEDIM), Università del Piemonte Orientale, Novara, Italy; Department of Health Care Management, School of Public Health, Tehran University of Medical Sciences, Tehran, Iran; Research Center in Emergency and Disaster Medicine and Computer Science applied to Medicine (CRIMEDIM), Università del Piemonte Orientale, Novara, Italy

## Abstract

Disaster education needs innovative educational methods to be more effective compared to traditional approaches. This can be done by using virtual simulation method. This article presents an experience about using virtual simulation methods to teach health professional on disaster medicine in Iran.

The workshop on the "Application of New Technologies in Disaster Management Simulation" was held in Tehran in January 2015. It was co-organized by the Disaster and Emergency Health Academy of Tehran University of Medical Sciences and Emergency and the Research Center in Disaster Medicine and Computer Science applied to Medicine (CRIMEDIM), Università del Piemonte Orientale. Different simulators were used by the participants, who were from the health system and other relevant fields, both inside and outside Iran.

As a result of the workshop, all the concerned stakeholders are called on to support this new initiative of incorporating virtual training and exercise simulation in the field of disaster medicine, so that its professionals are endowed with field-based and practical skills in Iran and elsewhere.

Virtual simulation technology is recommended to be used in education of disaster management. This requires capacity building of instructors, and provision of technologies. International collaboration can facilitate this process. Keywords: Virtual simulation, disaster management, education, training, Iran

## Introduction

The Islamic Republic of Iran (I.R.Iran) has been always exposed to disasters. The 2003 Bam earthquake accelerated the impetus of formulating effective disaster response and risk management actions in the whole disaster management and health systems of Iran.^1^^,^ 2 This revolution was supported by different stakeholders including government and academia. Thereafter, consistent with new strategies on disaster management, the necessity of relevant training initiatives was considered, in which the Master of Public Health (MPH) with disaster concentration, and the PhD program of Disaster and Emergency Health were developed in Iran.^3^ Although this initiative consists of academic and field approaches, in respect to training and exercise interventions, there is high demand to import new educational technologies, with the aid of virtual training and simulation in the subject of disaster management. To fulfill this goal, the first workshop on “Application of New Technologies in Disaster Management Simulation” was organized by the Disaster and Emergency Health Academy of Tehran University of Medical Sciences (TUMS) in collaboration with the Research Center in Emergency and Disaster Medicine and Computer Science applied to Medicine (CRIMEDIM), Università del Piemonte Orientale, Italy.

## Virtual simulation

In the past two decades, simulation has been used increasingly as part of the ongoing evolution of technology. Disaster medicine is among the fields where different simulation models have been integrated. The term "Simulator", in relation to healthcare, refers to a device that presents a simulated environment, patient, and situations, and then interacts appropriately with the actions taken by simulation participant.^4^ Although the history of simulation techniques predates information technology, using computerized simulators has brought huge capabilities in producing a life-like world for instructors and trainees.^5^ The Society for Academic Emergency Medicine Simulation Task Force, established in 2005, has been keen to promote virtual simulation awareness and its incorporation in emergency medicine and healthcare.^6^ Such training provides students and researchers with opportunities to apply their medical theoretical knowledge in a safe and realistic setting, develop team working skills, and a systematic approach to problem solving.^7^ In this way, disaster medicine is a subject with a high potential to use computerized simulations tools and methods. In fact, due to ethical, logistic, and financial barriers, it is not easy to make a real simulated disaster situation for educational purposes. For instance, it is impossible to simulate a huge catastrophe like the Bam earthquake in training and exercise programs other than through computer simulation.

Within the past few years, there have been ongoing improvements in disaster exercises and simulations in the health system of Iran, but computerized simulation-related training benefits are yet to be appropriately realized and applied.

## First workshop on application of new technologies in disaster management simulation in Iran

From 3rd to 6th January 2015, the TUMS in collaboration with CRIMEDIM organized a training workshop on application of new technologies in disaster management simulation in Tehran. It attracted participants studying and working in the health disaster arena. The training aim was fourfold to:

- provide participants with the conceptual framework and methodological aspects of virtual reality simulation and identity skills and abilities necessary in carrying them out;
- design, develop and evaluate virtual reality simulations for emergencies and disasters;
- provide practical IT tools for evaluation of exercises and virtual reality simulations; and
- present good practices in medical management of real disasters, exercises and virtual reality simulations.

This four-day intensive program covered the following topics: incident scene management, triage and rescue operations; medical management of Chemical, Biological, Radiological and Nuclear (CBRN); management of medical systems and resources in pre-hospital and hospital operations; and evaluation of emergency exercises.

Aside from the presentations, with aid of the XVR Simulator® and ISEE Simulator® (E-Semble, Delft, The Netherlands)^4^, the trainers engaged participants in group based and tabletop computerized simulative exercises. These exercises mimic some mass causality incident (MCI) scenarios according to the training topics on triage and medical resource management, both in the pre-hospital and hospital setting. The simulation-based learning environment was created in accordance with the four criteria for designing effective simulations for learning procedural skills.^8^ At the end of the computerized exercises, structured debriefings were delivered based on the theoretical principles developed by Fanning and colleagues^9^, showing the objective data (i.e.: pre-hospital, in-hospital, dispatch center performance indicators^10^ and triage accuracy) easily gathered and presented with the use of the Disaster Simulation Suite – DSS® software (iNovaria, Novara, Italy).

As well as these commercial software, different free software was also demonstrated and used by participants, with the aim of training and exercise; for instance, TIER Threat Identification tool, TIER Risk Calculator, Electronic Mass Causality Assessment and Planning Scenarios (EMCAPS), and Virtual Community Reception Center (VCRC).^11^

At the workshop closure, almost all the participants’ evaluation comments called for more similar virtual simulation training opportunities across Iran given the mesmerizing and inspiring knowledge, skills and concepts they gained. For other participants, it was an opportunity to rehearse and identify their personal and institutional strengths and weaknesses in relation to their previous roles in some MCIs in Iran and elsewhere.

## A need to incorporate virtual simulation in disaster education in Iran

The unpredictable and multifaceted exposure of the I.R.Iran to disasters warrants an urgent effort to endow disaster scholars and professionals with more practical skills rather than theoretical ones. Ideally, this can be fostered by exploiting virtual simulation training. With the recent initiative of the TUMS to organize training on new technologies in disaster management simulation, it is evident that adoption of virtual simulation in Iran is at hand. As a first step, this can be done most effectively by: conducting numerous and rapid countrywide consultations with various disaster professionals in order to identify the potential learners; assess their general and specific simulation educational needs; define learning objectives; and select the best teaching and training strategy for achieving each objective.^5^ The experience gained from this assessment can guide the development of a comprehensive virtual simulation teaching and learning approach and later be incorporated into the Iranian professional and academic training curricula.

## Conclusion

With reflection on this assessment of the application of new technologies in disaster management simulation training in mind, we conclude by calling for support and extended collaboration within and outside Iran from all concerned to effectively incorporate virtual simulation with the ultimate goal of endowing disaster professionals with field-based and practical skills in Iran. The same call should also be made beyond Iran to other countries and regions known to be embroiled in devastating disasters.

## Competing Interest

Ali Ardalan, Joseph Kimuli Balikuddembe, and Ali Akbarisari have declared that no competing interests exist. Pier Luigi Ingrassia, Luca Carenzo, Francesco Della Corte, and Ahmadreza Djalali are working for CRIMEDIM. Pier Luigi Ingrassia, Luca Carenzo, and Francesco Della Corte, partially own iNovaria (Novara, Italy), the academic spinoff company of the Universita` degli Studi del Piemonte Orientale ‘A. Avogadro’, and the DSS software. iNovaria is the sales partner of E-Semble Company (Delft, the Netherlands), the owner of the XVR and ISEE software.

## Corresponding author

Ali Ardalan MD, PhD. Tehran University of Medical Sciences, Tehran, Iran; Harvard Humanitarian Initiative, Harvard University, Cambridge, USA. Email: aardalan@tums.ac.ir
